# Renin-Angiotensin System Hyperactivation Can Induce Inflammation and Retinal Neural Dysfunction

**DOI:** 10.1155/2012/581695

**Published:** 2012-03-22

**Authors:** Toshihide Kurihara, Yoko Ozawa, Susumu Ishida, Hideyuki Okano, Kazuo Tsubota

**Affiliations:** ^1^Department of Ophthalmology, Keio University School of Medicine, 35 Shinanomachi, Shinjyuku-ku, Tokyo 160-8582, Japan; ^2^Laboratory of Retinal Cell Biology, Keio University School of Medicine, 35 Shinanomachi, Shinjyuku-ku, Tokyo 160-8582, Japan; ^3^Department of Cell Biology, The Scripps Research Institute, MB 28, 10550 North Torrey Pines Road, La Jolla, CA 92037, USA; ^4^Department of Ophthalmology, Hokkaido University Graduate School of Medicine, N-15, W-7, Kita-ku, Sapporo 060-8638, Japan; ^5^Department of Physiology, Keio University School of Medicine, 35 Shinanomachi, Shinjyuku-ku, Tokyo 160-8582, Japan

## Abstract

The renin-angiotensin system (RAS) is a hormone system that has been classically known as a blood pressure regulator but is becoming well recognized as a proinflammatory mediator. In many diverse tissues, RAS pathway elements are also produced intrinsically, making it possible for tissues to respond more dynamically to systemic or local cues. While RAS is important for controlling normal inflammatory responses, hyperactivation of the pathway can cause neural dysfunction by inducing accelerated degradation of some neuronal proteins such as synaptophysin and by activating pathological glial responses. Chronic inflammation and oxidative stress are risk factors for high incidence vision-threatening diseases such as diabetic retinopathy (DR), age-related macular degeneration (AMD), and glaucoma. In fact, increasing evidence suggests that RAS inhibition may actually prevent progression of various ocular diseases including uveitis, DR, AMD, and glaucoma. Therefore, RAS inhibition may be a promising therapeutic approach to fine-tune inflammatory responses and to prevent or treat certain ocular and neurodegenerative diseases.

## 1. Introduction

Most visual disorders occur in the retina, which is a part of the central nervous system (CNS) and consists of neurons, glia, pigment epithelium (RPE), and blood vessels. Currently, diabetic retinopathy (DR), age-related macular degeneration (AMD), and glaucoma are the top causes of blindness in the developed countries. These diseases can occur when local or systemic neuronal and vascular homeostasis mechanisms are dysregulated. The highest risk factor for many of these diseases is aging [1–3], and as is the case with other age-related diseases such as Alzheimer's disease, cardiovascular disease, cancer, arthritis, osteoporosis, and hypertension, accumulating evidence suggests that chronic inflammation and oxidative stress can accelerate or promote disease progression [4–6].

The renin-angiotensin system (RAS) is classically known as a systemic blood-pressure-regulating system. However, it is becoming widely recognized as an inflammation regulator as well. Independent of systemic RAS, tissue intrinsic RASs have been identified in various tissues (including the retina) and are important for maintaining local homeostasis. Elements of the RAS pathway are highly conserved in many species including invertebrates and humans demonstrating that its functions are evolutionarily conserved, although spatial expression patterns differ slightly between different species [7].

We have reported that angiotensin II type 1 receptor blocker (ARB) suppresses retinal neural dysfunction in animal models of acute inflammation [8] or diabetes [9]. Other groups and our own have also reported that ARBs can protect retinal vascular inflammation [10–19] and neuronal apoptosis [20–23]. Furthermore, it was recently reported by two independent groups that daily oral administration of ARB may prevent the progression of diabetic retinopathy in randomized multicenter clinical trials [24–26]. In this paper we will summarize these findings and other studies demonstrating that RAS modulation may prevent ocular pathogenesis. We will also outline the similarities and differences between retinal and brain RAS. Lastly, we will describe the potential mechanisms through which RAS inhibition may preserve neuronal function and viability while combating ocular diseases.

## 2. RAS as an Inflammatory Cascade

**Figure 1:**
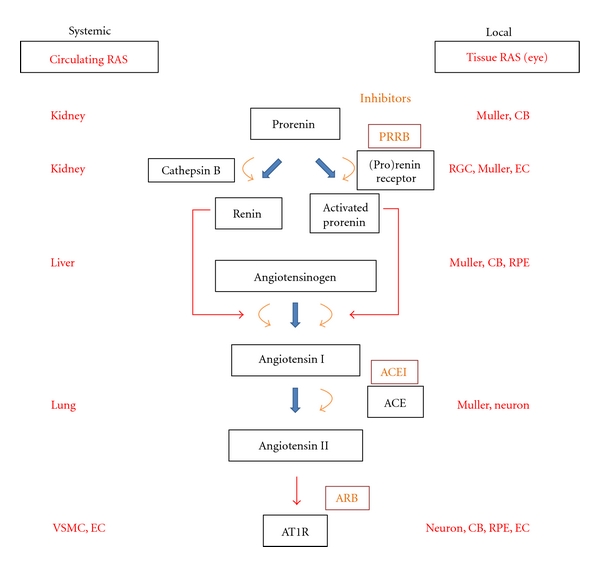
Circulating and tissue renin-angiotensin system (RAS). VSMC: vascular smooth muscle cell, EC: endothelial cell, PRRB: (pro)renin receptor blockers, ACEI: angiotensin-converting enzyme inhibitors, ARB: angiotensin II type 1 receptor blockers, AT1R: angiotensin II type 1 receptor, CB: ciliary body, RPE: retinal pigment epithelium.

Renin was discovered as a hypertensive agent in rabbit kidneys in 1898. It was later found to induce the release of a vasoconstrictive agent in experimental models of hypertension induced by renal ischemia [27]. Two independent groups identified the end product of this hypertensive cascade in 1939 and named it “hypertension” [28] or “angiotonin” [29]. It has since been renamed “angiotensin” [30]. The RAS pathway as we know it today began to take shape once angiotensin-converting enzyme (ACE) was identified in 1956 [31]. We now know that once renin is proteolytically processed from its precursor prorenin by proteases and released from the kidney, it converts angiotensinogen to angiotensin I in the liver. Angiotensin I is finally converted to angiotensin II by ACE which is predominantly expressed in vascular endothelial cells (ECs) and is located in highly vascularized tissues such as the lung (Figure 1). Angiotensin II stimulates vascular smooth muscle cells (VSMCs) that line endothelial cells to contract and induce vasoconstriction.

There are two primary receptors for angiotensin II: angiotensin II type 1 receptor (AT1R) and AT2R; AT1R appears to exert predominant functions in blood vessels. Generally, AT1R signaling normally induces vasoconstriction while AT2R signaling induces vasodilation. However, the roles of AT1R and AT2R in pathophysiological conditions are currently under debate [32–34]. AT1R is a seven-transmembrane G protein-coupled receptor [35, 36]. Once stimulated in VSMCs G proteins activate phospholipase C (PLC) and inositol-1,4,5-triphosphate (IP3) to open calcium channels in the endoplasmic reticulum [37]. As a result, increase of cytosolic calcium induces phosphorylation of myosin light chain, VSMC contraction, and vasoconstriction [38, 39].

Independent of systemically circulating angiotensin II (circulating RAS), most RAS components, including ACE, were also found to be locally expressed in many tissues [40]. This observation resulted in the hypothesis that in addition to being converted in particular organs for systemic circulation, angiotensin II could also be synthesized in peripheral tissues (tissue RAS) where it would exert its effect locally. Tissue RAS elements were identified in various organs including heart [41], kidney [42], adrenal gland [43], brain [44], and retina (see details below). An important molecule involved with tissue RAS is (pro)renin receptor which interacts with prorenin to exert enzymatic activity of renin without the conventional proteolysis of the prorenin prosegment [45, 46]. (Pro)renin receptor can be detected in major organs but not in circulation indicating that this molecule may play a critical role in the activation of tissue RAS [46]. Thus tissue RAS may be important for fine-tuning global RAS activity or for acting intrinsically to respond to localized insults. However, (pro)renin receptor may also function independent of renin activation as a member of the Wnt receptor complex to regulate Wnt/ß-catenin pathway activity [47].

In addition to its critical physiological functions, RAS dysregulation can lead to pathogenesis. In various cardiovascular cell-type RASs hyperactivation can induce pathogenic cell migration, hypertrophy, fibrosis, disrupt cell adhesion and ectopic extracellular matrix (ECM) deposition. AT1R signaling directly activates key signaling pathways for cell growth and hypertrophy including JAK/STAT (janus kinase/signal transducer and activator of transcription) [48, 49], ERK (extracellular-signal-regulated kinase) 1/2 [50–52], and p38 MAPK (mitogen-activated protein kinase) [53]. Indeed, angiotensin II/AT1R signaling can potentiate oxidative stresses and inflammatory responses by activation of NAD(P)H (nicotinamide adenine dinucleotide phosphate) oxidases [54–57]. Angiotensin II can also activate EGFR (epidermal growth factor receptors) and induces fibronectin synthesis and TGF*β* (transforming growth factor beta) activity to promote fibrosis and ECM formation [58, 59]. AT1R signaling can activate NF*κ*B (nuclear factor kappa-light-chain-enhancer of activated B cells) [60–62] and AP-1 (activator protein 1) to initiate transcription of multiple proinflammatory genes [61, 63, 64]. AT1R also induces accumulation, adhesion, and infiltration of inflammatory cells via activation of PAI-1 (plasminogen activator inhibitor-1) [65] and MCP-1 (monocyte chemotactic protein-1) [62] to promote atherosclerosis [66]. Taken together, these findings provide strong evidence that RAS is not just a regulator of blood pressure, but also regulates an inflammatory cascade.

The effects of circulating and tissue RAS can be controlled with RAS inhibitors. After the first ACE inhibitor (ACEI) was developed [67], many other RAS inhibitors including ARB [68, 69] have been established and approved for commercial use as hypertension drugs (Figure 1). RAS inhibition not only prevents hypertension but also protects tissues against injury by limiting the potency of deleterious inflammatory responses. Since aging is considered to be, in part, the result of chronic inflammation [70], it may not be too surprising that the use of RAS inhibitors or genetic deletion of AT1R has potential to extend the life span in hypertensive [71–73] or normotensive [74] mammals.

## 3. Brain and the Retinal RAS

In addition to regulating vasoconstriction, another important physiological function of RAS is osmoregulation in the CNS (e.g., water and sodium intake, sympathetic activity, and release of vasopressin) [75–77]. AT1R is expressed in brain neurons and mediates osmoregulation [76] by stimulating the release of vasopressin in the pituitary gland and signaling the kidney to conserve water [76]. Furthermore angiotensin II/AT1R signaling in the brain forces individuals to stimulate increased thirst and consume more drinking water [77]. Since angiotensin II has a high molecular weight, it does not cross the blood-brain barrier (BBB) [78]. Therefore intrinsic RAS networks must be responsible for inducing the dipsogenic activity. Additionally, every component of the RAS pathway including angiotensinogen, ACE, and angiotensin II receptors is expressed in the brain [75, 76, 79–81]. Brain RAS can also become dysregulated; this has been shown to induce oxidative stress and inflammation [82]. However, RAS inhibitors have neuroprotective effects in brain inflammation and ischemia without inducing antihypertension (see detail below).

The retina also has an intrinsic tissue RAS. In the eye, prorenin protein and renin activity can be detected in the vitreous fluid [83–85] and prorenin mRNA has been detected in Muller glia [86] and in the ciliary body (CB) cells [87]. (Pro)renin receptor is expressed in ECs, Muller glia, and retinal ganglion cells (RGCs) [88, 89]. Angiotensinogen is found in CB [90], Muller glia [91], and RPE [92]. ACE is synthesized in the neural retina [93, 94] and can be detected in RGCs, photoreceptors [95], and Muller glia [96]. Angiotensin II, the final product of RAS, can be detected in the vitreous fluid [97] and in the neural retina [98]. Interestingly, the normal concentration of angiotensin II in ocular fluid is higher than in plasma [97], confirming the existence of tissue RAS in the eye.

**Figure 2:**
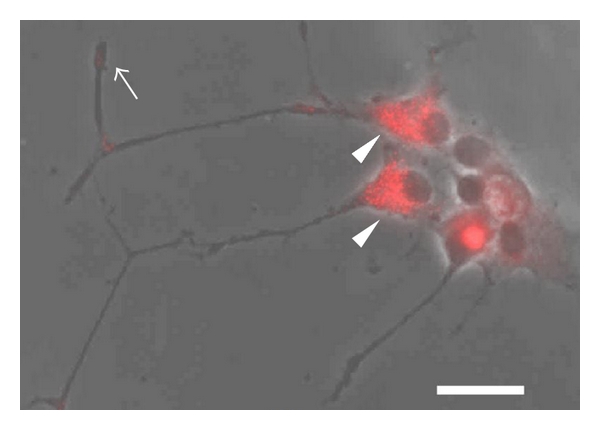
AT1R expression in a neuronal cell line. Immunohistochemistry for angiotensin II type 1 receptor (AT1R) in PC12D cells. Note that AT1R is detected in presynaptic terminal (arrow) or soma (arrow head). Scale bar: 20 *μ*m.

In the retina, angiotensin II receptors are detected both in ECs and in neuronal cells, which are located outside and inside of the blood-retina barrier (BRB), respectively [8, 92, 99, 100]. AT1R is found in the presynaptic terminals of photoreceptors and of interneurons in the retina [8] as well as in neurons of the brain [101, 102] (Figure 2). AT1R is also expressed in RGCs [103], although the physiological function of AT1R in the neural retina is not fully understood. Systemic administration of ACEI negatively influences cat and human neural functions measured by electroretinograms (ERG) in both systemic blood-pressure-dependent [104] and -independent manners [105, 106]. Additionally, angiotensin II increases voltage-dependent calcium currents in cultured RGCs [103]. Therefore ocular RAS may act as a physiological neuromodulator.

AT2R is also expressed in the retina [8] but much less is known how it functions in the eye. Polymorphisms in the AT2R gene may be linked to glaucoma [107] or diameter of the retinal arterioles [108].

## 4. RAS and Ocular Diseases

### 4.1. Uveitis

Increasing evidence suggests that RAS activity and inflammation may be associated with various ocular diseases, and, therefore, RAS inhibitors may be effective therapeutic agents. Several lines of evidences suggest that RAS inhibition is an effective treatment for uveitis [8, 12, 17, 18, 88]. Endotoxin-induced uveitis (EIU) is induced with intraperitoneal injections of lipopolysaccharide (LPS); this results in upregulated expression of proinflammatory and adhesion molecules such as ICAM-1 (intercellular adhesion molecule 1), MCP-1, IL-6 (interleukin 6), and IFN-*γ* (interferon-gamma) [17, 88]. These molecules are also upregulated in experimental autoimmune uveoretinitis (EAU) models generated by immunizing animals with interphotoreceptor retinoid-binding protein (IRBP) [18]. The upregulation of these molecules, however, can be inhibited with ARB or (pro)renin receptor blocker (PRRB). (PRRB is an experimental decoy peptide that contains “handle” region sequence of (pro)renin receptor.) RAS inhibition also suppresses retinal leukocyte stasis, CD4+ T-cell activation [17, 18, 88]. Furthermore, RAS inhibition suppresses gliosis by preventing STAT3 activation [8]. Lastly, when the expression levels of RAS pathway components are examined in EIU, prorenin, (pro)renin receptor [88], angiotensin II [8], and AT1R [17] levels are elevated in the retina. These findings suggest that heightened inflammatory responses in the eye and RAS activation are strongly correlated.

### 4.2. Chronic Inflammation and Eye Diseases

**Figure 3:**
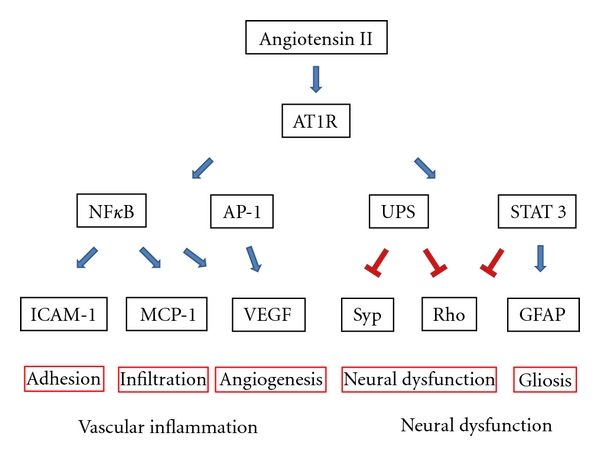
Downstream of AT1R in vascular inflammation and neural dysfunction. NF*κ*B: nuclear factor kappa-light-chain-enhancer of activated B cells, AP-1: activator protein 1, UPS: ubiquitin-proteasome system, STAT3: signal transducer and activator of transcription 3, ICAM-1: intercellular adhesion molecule 1, MCP-1: monocyte chemotactic protein 1, VEGF: vascular endothelial growth factor, Syp: synaptophysin, Rho: rhodopsin, GFAP: glial fibrillary acidic protein.

Besides being correlated with classically acute inflammation cases such as uveitis, one of the largest risk factors for developing prevalent and vision-threatening diseases such as DR, AMD, and glaucoma is aging [1–3]. These age-related eye diseases [109, 110] and others [5, 6] are now known to be caused (at least partially) by chronic inflammation and oxidative stress. Since RAS inhibition may prolong the life spans of hypertensive [71–73] or normotensive [74] mammals, it is logical that age-related eye diseases may be prevented or treated by suppressing inflammation and oxidative stress. The main pathological event of DR and AMD is abnormal neovascularization and VEGF (vascular endothelial growth factor) has been known to be a large contributor for them [111–113]. VEGF is a potent angiogenic factor and an inflammatory cytokine that induces the accumulation, adhesion, and infiltration of leukocytes [114, 115]. Inflammatory response in the retina can promote tissue ischemia by inducing vascular regression (vaso-obliteration) and also pathological angiogenesis [116]. Angiotensin II can induce upregulation of VEGF receptor (VEGFR)-2 and angiopoietin-2 in retinal ECs [117, 118] and VEGF in retinal pericytes [119] (Figure 3). Oxygen-induced retinopathy (OIR) is an animal model induced by continual aeration with 75–80% oxygen in early postnatal stages. OIR animals develop stereotypical phenotypes and is useful to evaluate vaso-obliteration and pathological angiogenesis (tuft formation) in the developing retina [120] which is largely regulated by VEGF [121]. This phenotype can be prevented with RAS inhibitors ACEI [122, 123], ARB [15], or PRRB [89, 124] that prevent pathological angiogenesis in OIR. The use of ARB and PRRB has the added benefit of suppressing abnormal angiogenesis without suppressing physiological vascular regeneration [15, 124]. In animals exposed to OIR RAS inhibitors may function to prevent gene expression of proinflammatory molecules and prevent leukocyte infiltration. Infiltration of VEGF-expressing inflammatory cells into the vitreous cavity is thought to induce pathological angiogenesis by causing ECs to grow in the wrong direction [115].

### 4.3. Diabetic Retinopathy (DR)

DR is one of the leading causes of blindness in the world [3]. It is characterized by vascular loss due to hyperglycemia and inflammation due to oxidative stress and AGEs (advanced glycation end products) accumulation. In severe cases hypoxia induces abnormal neovascularization (proliferative diabetic retinopathy, PDR) in addition to hyperpermeability (diabetic macular edema; DME). Prorenin [83] and angiotensin II [125, 126] are found to be increased in the vitreous humor of PDR and DR patients. RAS may potentiate the vascular phenotype of DR by upregulating VEGF/VEGFR-2 signaling (through angiotensin II) [118, 119] thereby inducing neovascularization and promoting blood vessel permeability. In fact, VEGF was initially named “vascular permeability factor” (VPF) [127].

Multiple attempts have been made to treat DR with RAS inhibitors. Although in one study ACEI administration seemed to attenuate retinal hyperpermeability in diabetic patients [128], interpretations of these studies are still being actively debated [129, 130]. However, recently three independent groups showed that ARB prevents BRB breakdown in animal models [131–133]. In 1998 and 2008, the results of randomized double-blind placebo-controlled trials using ACEI or ARB to treat DR were released from the EUCLID (EURODIAB Controlled Trial of Lisinopril in Insulin-Dependent Diabetes; ACEI treatment) [134] and DIRECT (Diabetic Retinopathy Candesartan Trial; ARB treatment) [24, 25]. Afterwards, RASS (Renin-Angiotensin System Study) in which both inhibitors were tested in DR patients was also released [26]. Large number of participants were examined in these trials (354 (type 1 diabetes) for EUCLID, 1421 (type 1) and 1905 (type 2) for DIRECT, and 285 (type 1) for RASS, resp.), and the results from all three studies provided strong evidence that RAS inhibition delays the onset or prevents the development of human DR symptom. However, these treatments were not universally beneficial. For example, in DIRECT, ARBs were not effective with respect to primary endpoints and had differing effects regarding secondary endpoints in different patient groups (type I or type II diabetes) [24, 25].

Clues for why RAS inhibition is effective for treating DR have come from animal studies. Streptozotocin (STZ) injections in rodents induce leukocyte stasis, blood vessel hyperpermeability, and formation of acellular capillaries. Importantly, ERG recordings are attenuated in rodents after STZ injections before vascular phenotypes are observed, indicating that neuronal dysfunction precedes neovascularization in diabetic models [9, 135]. Apoptosis of retinal neurons is also observed in later stage [136]. The administration of ACEI [137–140], ARB [10, 13, 14, 141], or PRRB [142] has been shown to rescue the vascular phenotypes of STZ-induced diabetic retinas. To generate another and more severe model of DR, Ren-2 transgenic rats (that have severe hypertension due to genetic knock-in of a mouse ren-2 renin gene [143]) can be injected with STZ. In these rats advanced vascular phenotypes are observed (including abnormal endothelial proliferation). Even in this model ACEI [144] or ARB [19, 145, 146] administration served as effective treatments. RAS inhibitors probably function by suppressing inflammatory cascades [10, 14] and by preventing oxidative stress [147] by limiting NF*κ*B and NAD(P)H activation. RAS inhibitors may also function to directly inhibit glucose accumulation into retinal cells by modulating GLUT-1 (glucose transporter 1) expression [148]. Furthermore, ARB was reported to influence the expression of glyoxalase I, a key regulator of AGEs [11]. Lastly, even though AT1R and AT2R are considered to have opposing functions AT2R inhibition may also effectively treat DR by suppressing VEGF and angiopoietin-2 expression levels in experimental retinopathies [33, 149].

### 4.4. Age-Related Macular Degeneration (AMD)

AMD is one of the leading causes of blindness especially in western countries. The greatest risk factors are aging and smoking [1], and the central phenotypes are choroidal neovascularization (CNV; wet AMD) and atrophy of photoreceptors and RPE cells (dry AMD). While no cure exists for dry AMD, wet AMD is currently treated with VEGF inhibitors [112, 113]. Inflammation exacerbates the wet AMD phenotype since infiltrating macrophages promote CNV formation [150–152]. Experimental CNV can be induced using laser coagulation to mechanically disrupt Bruch's membrane. The size of the laser-induced lesions after treatment with ACEI [153], ARB [16], and PRRB [154] is significantly reduced. Furthermore, AT1R-deficient mice are resistant to laser-induced CNV [154]. RAS inhibition may protect against CNV formation by inhibiting RAS activity and suppressing ERK signaling (directly with (pro)renin receptor-mediated intracellular signaling) [154].

RPE cells are positioned between the choroidal vasculature and photoreceptors and have function to maintain the visual (retinoid) cycle and to form a tight seal that prevents choroidal vessel invasion. Angiotensin II signaling in RPE cells increases abnormal production [155–157] and excessive turnover [158] of ECM via MMP (matrix metalloproteinase)-2 and -14 thereby weakening the seal that prevent choroidal EC invasion. These studies suggest that RAS inhibition may be an effective treatment for AMD as well as DR.

### 4.5. Glaucoma

Glaucoma is another age-related and high incidence ocular disease [2]. The feature of this disease is neurodegenerative of RGCs, but it can be caused by heterogeneous and complex mechanisms. One direct mechanism to induce RGC death is to increase the intraocular pressure (IOP). Studies devoted to developing new methods of controlling IOP are critical and ongoing. However, a subpopulation of glaucoma patients have normal IOP (normal tension glaucoma, NTG). This complicates the development of effective therapies since both forms are induced by seemingly separate mechanisms. Some RAS components including angiotensin II receptors are expressed in CB cells [90, 159, 160] that secrete aqueous humor and regulate IOP. Like other antihypertensive drugs such as calcium channel blockers, ACEI or ARB decreases IOP in humans and other primates [161–165] although IOP is considered to be regulated independently of systemic blood pressure. In an experimental model of high IOP and glaucoma, ARB treatments effectively suppress RGC death [23]. These findings suggest that RAS inhibition may be effective for treating glaucoma patients with high IOP.

## 5. RAS Inhibition Protects Brain and Retinal Neurons

Angiotensin II receptors are expressed inside and outside of the BBB [75, 76, 79–81] and the BRB [8, 92, 99, 100] indicating that both circulating and tissue RAS exist in the CNS, and if dysregulated, could elicit pathological effects. Indeed, RAS inhibition can attenuate the degree of inflammation in the brain and the eye [166, 167]. Inhibiting RAS can prevent experimental brain injuries induced by middle cerebral artery occlusion [168, 169] by suppressing vascular inflammation [170], including BBB breakdown [171], and/or regulating neural apoptosis directly [169]. Interestingly, AT2R is more highly expressed in developing neuronal tissues in vivo than in adult tissues [172] and AT2R stimulation promotes axonal regeneration of optic nerve [173] and minimizes formation of ischemia-induced cerebral lesions [174]. This suggests that ARB, which not only blocks AT1R but also causes angiotensin II to bind AT2R [175], may be an ideal drug for treating inflammatory diseases in the CNS. Inhibition of RAS may also prevent stress-induced behaviors including anxiety, depression, and panic by suppressing the release of corticotrophin-releasing factor [176–178]. Furthermore, recent studies suggest that brain RAS may potentiate Alzheimer's disease progression by stimulating the production of beta amyloid [179–182].

Retinal dysfunction as detected in ERG recordings can be observed in early diabetic animal models and in humans before vascular changes and neural cell loss are observed [135]. Amazingly, these deficits can be prevented by inhibiting RAS [9, 183, 184]. We have reported that ARB prevents retinal dysfunction (e.g., decrease of amplitude and an extension of the implicit time of ERG) in EIU [8] and in STZ-induced early diabetic retinas [9]. Furthermore, in these inflamed retinas, we determined that angiotensin II prompted the degradation of the presynaptic protein synaptophysin through the ubiquitin proteasome system (UPS) [8, 9]. UPS-mediated degradation of rhodopsin (part of the light-responsive complex in photoreceptors) can also be observed in EIU via STAT3 activation (which operates downstream of AT1R) [8, 185]. Additionally, STAT3 signaling serves as a negative regulator of rhodopsin in differentiating photoreceptors during retinal development [186, 187]. Thus, regulating angiotensin-II-induced protein degradation could serve as an important neuroprotective measure [188] (Figure 3).

Another target of inflammation is reactive glia including microglia, astrocytes, and Muller glia. Activated glia cause gliosis and alter proper neuronal morphology. Microglia are resident CNS myeloid-derived cells and mediate critical immune and inflammatory responses. AT1R signaling induces activation of microglia via NF*κ*B and AP-1 [189, 190]. GFAP (glial fibrillary acidic protein) is a differential and reactive marker of astrocyte and Muller glia, respectively, and its transcription is regulated by STAT3 activation [191]. The activation of astrocytes and Muller glia in experimental retinopathy can be prevented by ARB [8, 192] (Figure 3), although it is important to consider that the contributions of reactive glia can be context dependent [193].

IOP-independent RGC apoptosis can be observed in STZ-induced diabetes [136], after ischemia/reperfusion [194], after optic nerve crush [195], and after intraocular NMDA (*N*-methyl-D-aspartic acid) injections [196] in animal models. RGC loss in diabetic hypertensive models can be prevented by ARB which restores oxidative redox and mitochondrial functions [22]. ACEI or ARB also prevents RGC apoptosis in ischemia/reperfusion models by suppressing toxic oxidative stress [21]. ARB can also rescue dying amacrine cells in OIR [20]. Polymorphisms of RAS pathway genes are reported to be associated with brain infarction or its early lesion [197–199] and AT2R gene polymorphisms are reported to be associated with the risk of NTG [107]. These findings may indicate that RAS inhibitors may directly protect retinal neurons from apoptosis and further suggest that RAS inhibition may be useful for therapeutic treatments of IOP-independent glaucoma.

## 6. Conclusion

RAS, which has been classically known as blood pressure regulator, is becoming widely recognized as a proinflammatory mediator. Many age-related ocular diseases may be caused or exacerbated by chronic inflammation. Cells in the eye are responsive to circulating and tissue RAS and increasing evidence indicates that RAS inhibition may prevent various ocular diseases including uveitis, AMD, and glaucoma. Based on the findings from multiple clinical trials, RAS inhibitors are effective therapeutic agents for treating DR although the results of these studies must be examined critically since the inhibitors were not universally beneficial. Other groups including our own have shown that RAS inhibitors protect neurons from oxidative stress and apoptosis by preventing posttranslational ubiquitination of proteins critical for retinal functions. Although not mentioned previously in this paper, another new and exciting RAS inhibitor, aliskiren (a direct renin inhibitor), has been developed. It may actually mediate more robust vascular protection than either ACEI or ARB [200]. Therefore, work is underway to characterize existing RAS inhibitors and to develop novel inhibitors since they hold great promise for attenuating chronic inflammation and for treating multiple ocular and nonocular diseases.

## Abbreviations List (In Order of Their Appearance)

RAS: Renin-angiotensin system
DR: Diabetic retinopathy
AMD: Age-related macular degeneration
CNS: Central nervous system
RPE: Retinal pigment epithelium
ARB: Angiotensin II type 1 receptor blocker
ACE: Angiotensin-converting enzyme
EC: Vascular endothelial cell
VSMC: Vascular smooth muscle cell
AT1R: Angiotensin II type 1 receptor
IP3: Inositol-1,4,5-triphosphate
PLC: Phospholipase C
ECM: Extracellular matrix
JAK: Janus kinase
STAT: Signal transducer and activator of transcription
ERK: Extracellular-signal-regulated kinase
MAPK: Mitogen-activated protein kinase
NAD(P)H: Nicotinamide adenine dinucleotide phosphate
NF*κ*B: Nuclear factor kappa-light-chain-enhancer of activated B cells
AP-1: Activator protein 1
EGFR: Epidermal growth factor receptor
TGF*β*: Transforming growth factor beta
PAI-1: Plasminogen activator inhibitor-1
MCP-1: Monocyte chemotactic protein-1
ACEI: ACE inhibitor
BBB: Blood-brain barrier
CB: Ciliary body
RGC: Retinal ganglion cell
BRB: Blood-retina barrier
ERG: Electroretinogram
EIU: Endotoxin-induced uveitis
LPS: Lipopolysaccharide
ICAM-1: Intercellular adhesion molecule 1
IL-6: Interleukin 6
IFN-*γ*: Interferon-gamma
EAU: Experimental autoimmune uveoretinitis
IRBP: Interphotoreceptor retinoid-binding protein
PRRB: (Pro)renin receptor blocker
VEGF: Vascular endothelial growth factor
VEGFR: VEGF receptor
OIR: Oxygen-induced retinopathy
AGE: Advanced glycation end-product
PDR: Proliferative diabetic retinopathy
DME: Diabetic macular edema
EUCLID: EURODIAB Controlled Trial of Lisinopril in Insulin-Dependent Diabetes
DIRECT: Diabetic Retinopathy Candesartan Trial
RASS: Renin-Angiotensin System Study
STZ: Streptozotosin
GLUT-1: Glucose transporter 1
CNV: Choroidal neovascularization
MMP: Matrix metalloproteinase
IOP: Intraocular pressure
NTG: Normal tension glaucoma
GFAP: Glial fibrillary acidic protein
NMDA: N-methyl-D-aspartic acid.
